# The relationship between clinical nurses’ feelings of underqualification and work withdrawal behavior: the multiple mediating roles of self-disgust and self-efficacy

**DOI:** 10.3389/fpsyg.2025.1588917

**Published:** 2025-05-14

**Authors:** Ran Yang, Na Li, Yuxia Fan, Zhenhao Yuan, Cuixia Lin, Yan Shen, Linlin Yang

**Affiliations:** ^1^School of Nursing, Shandong University of Traditional Chinese Medicine, Jinan, China; ^2^Qingdao Mental Health Center, Qingdao, China; ^3^Weifang Hospital of Traditional Chinese Medicine, Weifang, China; ^4^School of Health, Shandong University of Traditional Chinese Medicine, Jinan, China; ^5^Jinan Maternal and Child Health Care Hospital, Jinan, China

**Keywords:** nurses, underqualification, work withdrawal behavior, self-disgust, self-efficacy, mediation analysis, nursing

## Abstract

**Purpose:**

The purpose of this study is to analyze the influence of three variables—feelings of underqualification, self-disgust, and self-efficacy—on work withdrawal behavior, with the aim of drawing attention to the mental health of nurses.

**Methods:**

A total of 300 nurses were recruited in Shandong Province, China for the survey. Participants completed the General Information Questionnaire, the Perceived Inadequate Qualifications Scale, the Work Withdrawal Behavior Scale, the Self-Disgust Scale, and the General Self-Efficacy Scale. The potential relationships between these variables were explored.

**Results:**

The work withdrawal behavior of nurses in this study is characterized as being at a lower middle level. Work withdrawal behavior was positively correlated with feelings of underqualification and self-disgust, while showing a negative correlation with self-efficacy. Self-disgust and self-efficacy indirectly influence perceived underqualification and work withdrawal behavior. Both the mediating effect and the chain mediating effect were found to be significant.

**Conclusion:**

Self-disgust and feelings of underqualification can aggravate the work withdrawal behavior of nurses, while improving self-efficacy can help reduce this behavior. Nursing managers should create opportunities for skill development, prioritize the physical and mental health of nurses, and take steps to minimize work withdrawal behavior, thereby improving the quality of clinical nursing.

## 1 Introduction

Nursing care plays an indispensable role in the treatment and recovery of patients. The National Nursing Career Development Plan for China (2021–2025) emphasizes that nursing is a critical component of healthcare, highlighting the need to strengthen nursing teams to promote high-quality nursing development (National Health Commission, 2022). According to WHO statistics, the global shortage of nurses is significant, reaching approximately 5.9 million and is expected to increase to 9 million by 2030 (WHO, 2020). By the end of 2023, there were about 5.63 million registered nurses in China, equivalent to only 4 registered nurses per 1,000 people, indicating a serious shortage (Bai, 2024). With the growing demand for nursing services, nurses must confront a high workload of clinical tasks, manage complex nurse–patient relationships, continuously learn new nursing operations and techniques, and balance conflicts between work and family life. Under these long-term high workloads, it is easy for nurses to experience work withdrawal behavior.

Work withdrawal behavior refers to employees’ attempts to escape high-intensity work pressure or disengage from their work environment, manifesting as psychological withdrawal and behavioral withdrawal (Hammer et al., 2003). The essence of the difference between the two lies in the different levels of occurrence. Psychological withdrawal behavior involves the separation of individual cognition and emotion, manifesting as a lax working state and turnover intention. In contrast, behavioral withdrawal is explicit, demonstrated through tardiness, early departures, escaping from work, and resignation, all of which directly impact nursing work. The study found that 20% of nurses globally experienced behavioral withdrawal behavior, with 27.7 and 45% of nurses in intensive care and emergency departments exhibiting psychological work withdrawal behavior (Xu et al., 2023; Ren et al., 2024). In China, 37.3% of nurses displayed psychological withdrawal behavior, while 13.7% exhibited moderate to high levels of behavioral withdrawal behavior (Xiong et al., 2023). Nurses who experienced COVID-19 are more likely to develop psychological issues such as anxiety, depression, and traumatic stress, which can lead to job burnout and work withdrawal behavior (Zhu et al., 2023). Such withdrawal behaviors can adversely impact nursing care, patient disease management, and the well-being of nurses. Research indicates that behaviors such as tardiness, premature departures, and disengagement result in decreased unit efficiency, lower patient satisfaction, and increased rates of nurse absenteeism and turnover, exacerbating the nursing shortage (Elliethey et al., 2024). Moreover, higher levels of work withdrawal behaviors are associated with declines in nurses’ professional well-being, increased burnout, decreased job satisfaction, and negative effects on both physical and mental health (Meng et al., 2023). Therefore, understanding the factors that influence nurses’ work withdrawal behaviors is crucial for effective nursing practice.

Stress coping theory posits that when facing a stressor, an individual cognitively evaluates the stressor or external environment, considering aspects such as harm, threat, and challenge. Based on these evaluations, individuals adopt either positive or negative coping styles according to their cognitive assessments and abilities, ultimately influencing the outcome. This process encompasses four components: the stressor, cognitive evaluations, coping, and the outcome (Lazarus and Folkman, 1984). Therefore, we can analyze the process leading to work withdrawal behavior through the lens of this theory.

Feelings of underqualification refer to the perception of a mismatch between an individual’s qualifications and their job requirements, occurring when an employee subjectively believes that their education, knowledge, experience, skills, or abilities are inadequate for their role (Sim and Lee, 2017). As a stressor, feelings of underqualification significantly affect the occurrence of nurses’ work withdrawal behavior. Due to the lack of unified education and guidance regarding role changes, new nurses in China often experience significant transition shock (Tao et al., 2023). In particular, new nurses facing high-intensity and challenging work may feel a pronounced sense of underqualification due to their limited work experience (Smythe and Carter, 2022). The pressure will cause nurses to have negative attitudes such as disgust, reduce their confidence in work, and eventually produce work withdrawal behavior. According to the Job Demands-Resources Model (Demerouti et al., 2001), excessive job demands combined with limited job resources can lead to employee burnout and withdrawal behavior. A qualitative study showed that the workload was heavy and there was not enough time to study, but the limited knowledge could not meet the wishes of patients, which further aggravated the feelings of underqualification and the withdrawal from work (Ericsson et al., 2022). Additionally, the introduction of advanced technologies into clinical practice can negatively impact nurses’ job satisfaction and their willingness to remain in their roles, particularly when feelings of underqualification are prevalent (Jedwab et al., 2023). Consequently, employees who experience heightened feelings of underqualification are more likely to exhibit work withdrawal behaviors (Liu, 2024).

Self-disgust is a persistent and recurring negative psychological condition that individuals, encompassing both the self and its behaviors (Ille et al., 2014; Overton et al., 2008). It involves a negative cognitive assessment of stressors. Under high work pressure, studies have shown that nurses often do not have enough time to master new knowledge and skills, which can lead to emotions such as anxiety and disgust, ultimately reducing their work engagement (Beaulieu et al., 2023). Additionally, the high risk of occupational exposure and perceived discrimination faced by specialized nursing groups, such as HIV nurses, can negatively impact their work-related emotions and mental health, potentially leading to feelings of aversion and withdrawal (Pan et al., 2022). Moreover, the low social status of Chinese nurses contributes to a diminished professional identity, which may further induce self-disgust and exacerbate work withdrawal behavior (Du et al., 2023). Influenced by a culture of collectivism, nurses often work within complementary roles. However, managing interpersonal relationships may create feelings of disgust and impair their ability to connect with and interact with others. Based on the available evidence, self-disgust may potentially be related to withdrawal behavior more generally and to social withdrawal (Murphy et al., 2021; Clarke et al., 2019). For example, nurses who have perfectionist tendencies toward themselves and others may lead to interpersonal conflict, feelings of inadequacy and psychological burdens that may can negatively impact their mood at work (Abdolkarimi et al., 2024). Nurses subjected to toxic leadership behaviors may develop self-doubt and disgust, undermining their confidence and potentially leading to work withdrawal behavior (Guo et al., 2022). In addition, nurses who experience workplace violence may encounter negative emotions, including self-disgust, guilt, and sadness, which can reduce their interactions with others, diminish their work interest, and lead to absenteeism (Zhang et al., 2022). Self-disgust is currently unexplored in the nurse population, and the relationship between self-disgust and work withdrawal behaviors needs further study.

Self-efficacy refers to a nurse’s perceived ability to cope with various stressors encountered in clinical work and their confidence in problem-solving, representing a positive coping strategy (Molero Jurado et al., 2019). In China, work stress, job burnout, and a lack of social support negatively impact nurses’ self-efficacy (Liu and Aungsuroch, 2019). Studies have shown a negative correlation between work stress and self-efficacy (Skogevall et al., 2022). For example, nurses who lack knowledge about venous thromboembolism (Venous thromboembolism is a disorder of impaired venous return in which blood clots abnormally in the veins, causing complete or incomplete blockage of the vessels.) often lack confidence in thromboembolism prophylaxis, leading to high levels of psychological stress and avoidance behaviors (Al-Mugheed and Bayraktar, 2023). According to self-efficacy theory, people with high self-efficacy will face pressure with a more positive attitude, relieve stressors and negative emotions, and reduce the occurrence of work withdrawal behavior (Bandura, 1977). Research indicates a significant positive correlation between nurses’ self-efficacy and work engagement; the higher the self-efficacy, the less likely they are to withdraw from work (Al-Hamdan et al., 2022). Additionally, as a positive coping strategy, self-efficacy can help adjust cognitive responses and effectively mitigate negative emotions (Huang et al., 2024). For example, self-efficacy can effectively alleviate negative feelings such as guilt and shame that arise during nursing work (Sarwar et al., 2021). Taken together, this suggests that self-efficacy may interact with other variables, although the exact nature of this interaction has not been fully explored.

**Figure 1:**
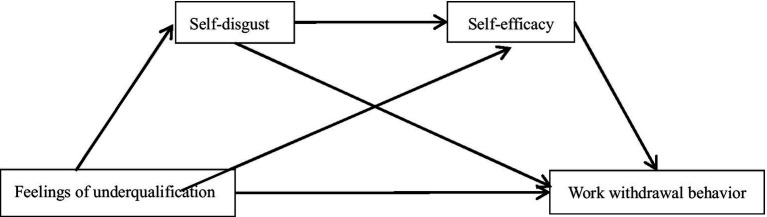
Hypothesized model: multiple mediating roles of self-disgust and self-efficacy between feelings of underqualification and work withdrawal behaviors.

This study utilizes stress and coping theory, with feelings of underqualification defined as the stressor, self-disgust as the cognitive assessment, self-efficacy as the coping style, and work withdrawal behavior as the outcome. The objective is to explore the effect of the independent variable—feelings of underqualification—on the dependent variable—work withdrawal behavior—along with the mediating effects of the other two variables. Currently, there are fewer studies on the effects of feelings of underqualification on work withdrawal behavior, and there is a dearth of research on the relationship and path of action of self-disgust and self-efficacy with feelings of underqualification and work withdrawal behavior. Therefore, the following hypotheses are proposed in this study (see Figure 1): (1) There is a correlation between feelings of underqualification and work withdrawal behavior; (2) Self-disgust mediates the relationship between feelings of underqualification and work withdrawal behavior; (3) Self-efficacy mediates the relationship between feelings of underqualification and work withdrawal behavior; (4) Self-disgust and self-efficacy have multiple mediating roles in the relationship between feelings of underqualification and work withdrawal behavior.

## 2 Methods

### 2.1 Design and participants

A cross-sectional study design utilizing convenience sampling was employed, and the STROBE checklist was followed to report the results. The study recruited participants from three hospitals in Shandong, China, between August 2024 and November 2024. The inclusion criteria for participants were as follows: (1) possession of a valid nursing license and registration with the hospital; (2) active engagement in clinical nursing; and (3) voluntary participation in the study. The exclusion criteria included: (1) nurses and nursing interns from external hospitals participating in further training; and (2) nurses who were not present during the survey period due to reasons such as attending further training, sick leave, or maternity leave.

According to Kendall’s principle of sample estimation (Wang, 2014), the required sample size is 10 times the number of items. This study includes a total of 21 independent variables, comprising 15 items in the General Information Questionnaire, 1 item related to feelings of underqualification, 2 items concerning self-disgust, 1 item on general self-efficacy, and 2 items regarding work withdrawal behaviors. Therefore, the calculated sample size is 210. Considering a 20% rate of invalid questionnaires, the estimated sample size was adjusted to 252. In this study, there were about 1,200 registered nurses in three hospitals. A total of 305 nurses were investigated, and 300 valid questionnaires were collected, with an effective recovery rate of 98.36%.

### 2.2 Measures

#### 2.2.1 Demographic characteristics

The General Information Questionnaire was developed by the research team and includes various demographic and professional variables of the participants. These variables comprise gender, age, education level, marital status, hospital grade, monthly income, employment relationship, title, working department, years of service, working hours, number of night shifts, mastery of clinical knowledge and skills, self-assessment of work stress, and clinical adaptability. In total, the questionnaire includes 15 items.

#### 2.2.2 Inadequate qualification scale

The Chinese version of the Inadequate Qualification Scale, originally developed by Johnson and Johnson (1996) and translated by Liu (2024), was utilized to measure nurses’ perceptions of self-qualification inadequacies. This scale is publicly available and widely used in research. It employs a 5-point Likert scale, where a score of 1 indicates “not at all” and a score of 5 denotes “completely,” resulting in a total possible score range of 4 to 20. Higher scores indicate a stronger perception of underqualification. Liu (2024) reported acceptable internal consistency (*α* = 0.927). In this study, the Cronbach’s alpha coefficient for the scale was 0.877.

#### 2.2.3 Work withdrawal behavior scale

The Chinese version of the Work Withdrawal Behavior Scale, developed by Lehman and Simpson (1992) and adapted by Yang (2016), was used to assess the work withdrawal behaviors of nurses. This scale is publicly available and widely utilized in research. It comprises two dimensions: psychological withdrawal and behavioral withdrawal, with a total of 12 items. The scale is based on a 5-point Likert scale, where 1 indicates “never” and 5 indicates “always.” The total score ranges from 12 to 60, with higher scores reflecting more frequent work withdrawal behaviors among nurses. Yang (2016) reported composite reliability of the scale was 0.840. In this study, the Cronbach’s alpha coefficient for the scale was 0.876.

#### 2.2.4 Self-disgust scale

The Chinese version of the Self-Disgust Scale, developed by Schienle et al. (2014) and adapted by Jin et al. (2016), was used to measure nurses’ feelings of self-disgust. This scale is publicly available and widely utilized in research. It consists of two dimensions: personal disgust and behavioral disgust, with a total of 14 items. The scale is scored on a 5-point Likert scale, where a score of 0 indicates “very uncomfortable” and a score of 4 indicates “very comfortable.” The total score ranges from 0 to 56, with higher scores reflecting higher levels of self-loathing. Jin et al. (2016) reported acceptable internal consistency (*α* = 0.895). In this study, the Cronbach’s alpha coefficient for the scale was 0.931.

#### 2.2.5 General self-efficacy scale

The Chinese version of the General Self-Efficacy Scale, developed by Schwarzer and Baessler (1996) and adapted by Wang et al. (2001). This scale was used to measure nurses’ self-efficacy because it was publicly and widely used and there was no recognized scale for nurses’ self-efficacy. It is unidimensional, consisting of 10 items, and is scored on a 4-point Likert scale, where 1 indicates “extremely uncomfortable” and 4 indicates “extremely comfortable.” The total score ranges from 10 to 40 points, with higher scores reflecting higher levels of self-efficacy. Wang et al. (2001) reported acceptable internal consistency (α = 0.927). In this study, the Cronbach’s alpha coefficient for the scale was 0.828.

### 2.3 Data collection

After obtaining ethical approval from the hospital as well as consent from the nursing department and the head nurse, the research team distributed the questionnaire electronically via the “Questionnaire Star” platform to participants who met the inclusion criteria. To ensure the quality of questionnaire collection, we referred to relevant studies by Regmi et al. (2016). Before initiating the study, the research team explained the study’s purpose and procedures to the participants, commencing questionnaire administration only after obtaining their informed consent. To assess the face validity of the questionnaire, we drew on the methodology of Allen et al. (2023). The design stipulated that the questionnaire could be answered only once per device. After completion, 15 subjects were randomly selected to provide feedback on the scale. They demonstrated a good understanding of the items, and the questions did not elicit discomfort. Thus, the selected scale was determined to have strong face validity. The research team reviewed the questionnaires on the spot and excluded those with obvious contradictions or patterns of response.

### 2.4 Ethics considerations

This study was approved by the Ethics Committee of Jinan Maternal and Child Health Hospital (No. KY R-24-147). In accordance with the Declaration of Helsinki, all participants provided informed consent to participate in the study after detailed information about the objectives, significance, potential risks and benefits of the study. And handle the data strictly in accordance with the theoretical standards of privacy protection (Zhang et al., 2025).

### 2.5 Statistical analysis

Data were processed and analyzed using SPSS version 27.0, and common method bias was assessed through Harman’s one-way test (Podsakoff et al., 2003). Demographic characteristics were analyzed with descriptive statistics. Independent samples *t*-tests and one-way ANOVA were employed to examine differences in demographic information related to work withdrawal behavior. Pearson correlation analysis was used to evaluate the relationships between bivariate variables, and variables with *p* < 0.05 in univariate analyses were included in multilevel regression analyses to assess the potential effects of feelings of underqualification, self-disgust, and self-efficacy on work withdrawal behavior. Chained mediation tests were conducted using Model 6 (Bolin, 2014) in the PROCESS macro, with significance tests for indirect effects performed using the bootstrap method and 95% confidence intervals (5,000 bootstrap samples). Mediation effects were considered significant if the 95% confidence intervals did not include zero.

## 3 Results

### 3.1 Common method bias test

All variables in this study were analyzed using exploratory factor analysis and assessed for common method bias through Harman’s one-way test. The results indicated that a total of six factors had eigenvalues greater than 1. The first factor explained 39.61% of the total variance, which is just below the 40% threshold. Therefore, there are no significant common method biases present in the data used in this study.

### 3.2 Socio-demographic characteristics

A total of 300 clinical nurses were included in this study, predominantly from secondary hospitals (59.3%). The mean age of the participants was 32.51 years (SD = 7.00 years), with 251 females (83.7%) and 49 males (16.3%). Most participants held a bachelor’s degree (75.7%) and had 11–20 years of work experience (37.3%), with the majority working as nurse supervisors (43.0%). Contractual employment was the most common employment type (42.3%).

The results of the independent samples t-test and one-way ANOVA revealed significant differences in work withdrawal behavior concerning several factors: gender (*t*[58.804] = 2.181, *p* = 0.033), literacy (*F*[3,296] = 3.030, *p* = 0.030), years of experience (*F*[4,295] = 2.447, *p* = 0.047), job title (*F*[3,296] = 7.764, *p* < 0.001), knowledge and skill acquisition (*F*[3,296] = 5.544, *p* = 0.001), job stress (*F*[4,295] = 7.160, *p* < 0.001), and clinical adaptability (*F*[4,295] = 18.163, *p* < 0.001). Statistically significant results were included as control variables in subsequent regression and mediation analyses to minimize their influence on the independent and mediating variables, thereby providing preliminary support for validating other hypotheses. Detailed results of the univariate analysis are presented in Table 1. The LSD results are shown in Table 2.

**Table 1:** Univariate analysis of variance between demographic information and work withdrawal behavior (*n* = 300).

	Work withdrawal behavior			
Variables	*N(%)*	*Mean ± SD*	*t/F*	*P*
Sex			2.181	0.033
Male	49(16.3%)	25.06 ± 9.22		
Female	251(83.7%)	22.04 ± 6.86		
Age groups			1.875	0.155
20–29 years	113(37.7%)	23.09 ± 7.91		
30–39 years	145(48.3%)	21.72 ± 6.62		
40 and above	42(14%)	23.83 ± 8.13		
Education level			3.030	0.030
Secondary school	12(4%)	27.42 ± 6.64		
High School	42(14%)	23.10 ± 7.39		
Undergraduate	227(75.7%)	21.96 ± 7.23		
Master’s degree and above	19(6.3%)	24.95 ± 7.86		
Marital status			1.338	0.262
Unmarried	71(23.7%)	23.99 ± 7.99		
Married	222(74%)	22.07 ± 7.14		
Divorced	4(1.3%)	21.00 ± 7.07		
Widowed	3(1%)	24.33 ± 8.33		
Hospital level			1.912	0.057
Triple A Hospital	122(40.7%)	23.51 ± 7.66		
Second-class hospitals	178(59.3%)	21.86 ± 7.11		
Monthly personal income			0.668	0.572
<3,000	35(11.7%)	23.97 ± 7.73		
3,000 ~ <5,000	87(29%)	22.53 ± 7.82		
5,000 ~ <8,000	130(43.3%)	22.46 ± 7.41		
≥8,000	48(16%)	22.67 ± 6.09		
Years of working experience			2.447	0.047
0–1 years	40(13.3%)	25.55 ± 8.25		
2–5 years	49(16.3%)	22.00 ± 8.54		
6–10 years	77(25.7%)	22.05 ± 7.02		
11–20 years	112(37.3%)	21.71 ± 6.18		
>20 years	22(7.3%)	24.05 ± 8.65		
Title			7.764	<0.001
Nurse	85(28.3%)	25.54 ± 8.53		
Nurse Practitioner	71(23.7%)	21.17 ± 7.01		
Nurse-in-charge	129(43%)	21.13 ± 5.88		
Vice professor of nursing and above	15(5%)	23.93 ± 8.72		
Employment relationship			1.894	0.152
Contract	127(42.3%)	23.20 ± 7.95		
Personnel agency	89(29.7%)	21.28 ± 6.42		
Regularly on board	84(28%)	22.83 ± 7.31		
Departments			1.402	0.233
Internal medicine	99(33%)	22.61 ± 7.99		
surgery department	46(15.3%)	23.17 ± 6.77		
Gynecology department	5(1.7%)	28.20 ± 8.64		
pediatrics department	46(15.3%)	23.28 ± 8.13		
Emergency department, operating room, ICU	104(34.7%)	21.57 ± 6.50		
Duration of work			−1.056	0.292
≤8 h	114(38%)	21.96 ± 8.11		
>8 h	186(62%)	22.88 ± 6.87		
Night shifts/month			0.327	0.721
≤4 times	93(31%)	22.04 ± 6.92		
5–10 times	185(61.7%)	22.70 ± 7.67		
>10 times	22(7.3%)	23.14 ± 6.77		
Knowledge and skill acquisition			5.544	0.001
Understanding	45(15%)	25.67 ± 8.69		
Familiarity with	83(27.7%)	23.61 ± 7.70		
Mastery	129(43%)	21.03 ± 6.34		
Proficient	43(14.3%)	21.65 ± 6.97		
Work pressure			7.160	<0.001
Very small	14(4.7%)	25.79 ± 12.32		
Smaller	20(6.7%)	21.15 ± 4.73		
Average	144(48%)	20.58 ± 6.34		
Large	106(35.3%)	24.26 ± 7.33		
Very large	16(5.3%)	27.44 ± 8.20		
Clinical adaptability			18.163	<0.001
Very poor	3(1%)	39.67 ± 14.98		
Poor	14(4.7%)	31.21 ± 7.36		
Fair	83(27.7%)	24.63 ± 7.36		
Good	158(52.7%)	21.43 ± 6.13		
Very good	42(14%)	18.40 ± 6.09		

**Table 2:** One-way analysis of variance was used for pairwise comparison (*n* = 300).

Variables	(I)	(J)	*MD* (I-J)	*SE*	*p*-value	95% *C I* (Lower, Upper)
Education level	Secondary school	Undergraduate	0.740	0.293	0.012	0.163, 1.317
Years of working experience	0–1 years	2–5 years	0.482	0.211	0.023	0.066, 0.897
		6–10 years	0.475	0.193	0.014	0.095, 0.855
		11–20 years	0.521	0.182	0.005	0.162, 0.880
		>20 years	0.204	0.263	0.438	−0.313, 0.722
	2–5 years	6–10 years	−0.007	0.181	0.969	−0.363, 0.349
		11–20 years	0.039	0.170	0.819	−0.295, 0.373
		>20 years	−0.278	0.254	0.276	−0.778, 0.223
	6–10 years	11–20 years	0.046	0.147	0.755	−0.243, 0.334
		>20 years	−0.271	0.239	0.259	−0.742, 0.201
	11–20 years	>20 years	−0.316	0.231	0.172	−0.771, 0.138
Title	Nurse	Nurse Practitioner	0.593	0.156	<0.001	0.287, 0.900
		Nurse-in-charge	0.598	0.135	<0.001	0.332, 0.865
Knowledge and skill acquisition	Understanding	Mastery	0.629	0.169	<0.001	0.296, 0.962
		Proficient	0.545	0.209	0.009	0.135, 0.955
	Familiarity with	Mastery	0.351	0.138	0.011	0.080, 0.621
Work pressure	Very small	Average	0.706	0.269	0.009	0.177, 1.236
	Smaller	Very large	−0.853	0.322	0.009	−1.488, −0.219
	Average	Large	−0.500	0.123	<0.001	−0.742, −0.257
		Very large	−0.930	0.253	<0.001	−1.429, −0.432

### 3.3 Correlation analysis

Following the first hypothesis of this study, we examined the correlation between the variables. Pearson’s correlation analysis revealed significant bivariate correlations between all variables. There was a significant positive correlation between feelings of underqualification and work withdrawal behavior (*r* = 0.777, *p* < 0.01) and self-disgust (*r* = 0.680, *p* < 0.01). Additionally, a significant positive correlation was observed between feelings of self-efficacy and work withdrawal behavior (*r* = 0.734, *p* < 0.01). Conversely, feelings of self-efficacy showed significant negative correlations with feelings of underqualification (*r* = −0.680, *p* < 0.01), work withdrawal behavior (*r* = −0.772, *p* < 0.01), and self-disgust (*r* = −0.702, *p* < 0.01). It not only verified the validity of Hypothesis 1 but also demonstrated the correlation between other variables, thereby laying the foundation for subsequent hypotheses. The specific results are shown in Table 3.

**Table 3:** Correlation between study variables (*n* = 300).

	Scale scores	Correlation analysis			
Variables	*Mean(SD)*	1	2	3	4
1. Feelings of underqualification	9.53 (3.93)	1			
2. Self-disgust	15.55 (10.19)	0.680^**^	1		
3. Self-efficacy Work	28.68 (5.67)	−0.765^**^	−0.702^**^	1	
4. Withdrawal behavior	22.53 (7.37)	0.777^**^	0.734^**^	−0.772^**^	1

SD, standard deviation. ** Significant correlation at the 0.01 level (two-tailed).

### 3.4 Hierarchical multiple regression analysis

According to the second and third hypotheses of this study, we performed multiple hierarchical multiple regression analyses. Statistically significant demographic information from the one-way analysis of variance was included in Model 1 as a control variable, explaining 21.4% of the variance in work withdrawal behavior. When the independent variable, perception of underqualification, was added to Model 2, it explained 64.6% of the variance and served as a significant positive predictor of work withdrawal behavior (*B* = 1.329, *p* < 0.001). Introducing the mediating variable, self-disgust, into Model 3 explained 69.6% of the variance, and the partial regression coefficient (*B*) between feelings of underqualification and work withdrawal behavior decreased from 1.329 (*p* < 0.001) in Model 2 to 0.974 (*p* < 0.001) in Model 3, indicating that self-disgust may partially mediate the relationship between feelings of underqualification and work withdrawal behavior. Hypothesis 2 was preliminarily verified. Finally, adding the mediating variable self-efficacy to Model 4 explained 72.8% of the variance, and the partial regression coefficient (*B*) further decreased to 0.672 (*p* < 0.001), suggesting that self-efficacy may also partially mediate the relationship between feelings of underqualification and work withdrawal behavior. Hypothesis 3 was preliminarily verified. The results of the hierarchical multiple regression analysis of work withdrawal behavior are presented in Table 4.

**Table 4:** Multiple linear regression analysis of work withdrawal behavior (*n* = 300).

	Work withdrawal behavior							
	Model 1		Model 2		Model 3		Model 4	
Variables	*B*	*P*	*B*	*P*	*B*	*P*	*B*	*P*
Sex	−3.109	0.003	−1.529	0.032	−1.36	0.04	−1.203	0.055
Education level	−0.571	0.432	0.337	0.492	0.579	0.204	0.782	0.072
Years of Working experience	0.69	0.144	0.989	0.002	0.869	0.003	0.829	0.003
Title	−0.943	0.145	−1.311	0.003	−1.035	0.011	−0.937	0.015
Knowledge and skill acquisition	−0.422	0.369	−0.228	0.469	0.055	0.851	0.068	0.808
Work pressure	0.827	0.073	−0.151	0.630	−0.369	0.207	−0.506	0.069
Clinical adaptability	−3.65	<0.001	−1.645	<0.001	−1.044	0.003	−0.959	0.005
Feelings of underqualification			1.329	<0.001	0.974	<0.001	0.672	<0.001
Self-disgust					0.239	<0.001	0.174	<0.001
Self-efficacy							−0.393	<0.001
*R^2^*	0.232		0.656		0.705		0.737	
*∆R^2^*	0.214		0.646		0.696		0.728	
*F*	12.602		69.253		77.02		80.846	

### 3.5 Chain mediation analysis

To test the fourth hypothesis of this study and to retest Hypotheses 2 and 3, we examined the mediating effects of self-disgust and self-efficacy on the relationship between feelings of underqualification and work withdrawal behavior using PROCESS macro Model 6. Gender, literacy, years of experience, job title, knowledge and skill acquisition, job stress, and clinical adaptability were included as control variables. First, we found that feelings of underqualification had a significant positive effect on self-disgust (*B* = 0.680, *p* < 0.001) and a significant negative effect on self-efficacy (*B* = −0.535, *p* < 0.001). Second, self-disgust exerted a significant negative effect on self-efficacy (*B* = −0.338, *p* < 0.001). Finally, both feelings of underqualification (*B* = 0.352, *p* < 0.001) and self-disgust (*B* = 0.279, *p* < 0.001) had a significant positive effect on work withdrawal behavior, while self-efficacy (*B* = −0.308, *p* < 0.001) demonstrated a significant negative effect on work withdrawal behavior.

**Figure 2:**
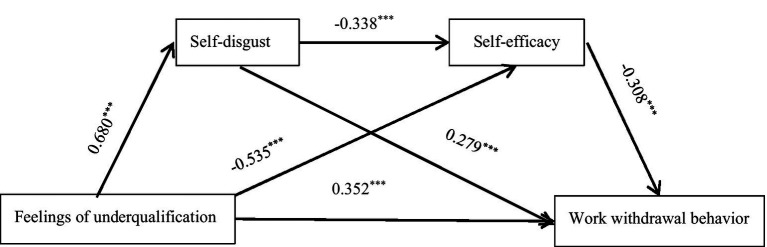
Mediation model of self-disgust and self-efficacy between feelings of underqualification and work withdrawal behavior. ^***^p < 0.001. Feelings of underqualification has a significant positive effect on self-disgust and work withdrawal behavior, and a significant negative effect on self-efficacy. Self-disgust had a significant negative effect on self-efficacy and a significant positive effect on work withdrawal behavior. Self-efficacy has a significant negative impact on work withdrawal behavior.

The total effect of feelings of underqualification on work withdrawal behavior was 0.777, while the direct effect was 0.352. The confidence intervals for each path do not include zero, indicating that both the mediation effect and the chain mediation effect are significant. The indirect effect of feelings of underqualification on work withdrawal behavior through self-disgust was 24.7%, thereby confirming Hypothesis 2. The indirect effect of feelings of underqualification on work withdrawal behavior through self-efficacy was 21.2%, which further verified Hypothesis 3. Additionally, the indirect effect of feelings of underqualification on work withdrawal behavior through self-disgust and self-efficacy was 9.1%, confirming Hypothesis 4. Specific results are presented in Table 5 and Figure 2.

**Table 5:** Direct and indirect effects between feelings of underqualification and work withdrawal behavior (*n* = 300).

Variables	Effect	BootSE	LLCI	ULCI	Ratio of indirect to total effect	Ratio of indirect to direct effect
Total effect	0.777	0.037	0.705	0.848		
Direct effect	0.352	0.506	0.252	0.451		
Total indirect effect	0.425	0.052	0.328	0.527	0.547	1.207
Ind1	0.190	0.047	0.101	0.288	0.247	0.540
Ind2	0.165	0.034	0.103	0.234	0.212	0.469
Ind3	0.071	0.018	0.039	0.111	0.091	0.202

Ind1, feelings of underqualification→ self-disgust → work withdrawal behavior. Ind2, feelings of underqualification→ self-efficacy → work withdrawal behavior. Ind3, feelings of underqualification → self-disgust → self-efficacy →work withdrawal behavior.

## 4 Discussion

In the present study, we explored the relationship between feelings of underqualification and work withdrawal behaviors, focusing on the mediating roles of self-disgust and self-efficacy. Consistent with our hypothesis, feelings of underqualification not only directly influence the onset of work withdrawal behavior in clinical nurses but also indirectly affect it through individual mediation by self-disgust and self-efficacy, as well as their combined interlocking effects. This cross-sectional study constructs an interlocking mediation model to examine the structural relationship between perceived underqualification and work withdrawal behavior. The results contribute to a deeper understanding of the mechanisms influencing nurses’ work withdrawal behaviors and further validate the overall coping process and its outcomes within the stress coping model. Based on these findings, targeted interventions can be developed to reduce the incidence of work withdrawal behaviors.

The mean score for work withdrawal behavior in this study was 22.53 (*SD* = 7.37), which aligns with the results of a previous study (Wang et al., 2024) and indicates a moderately low level of withdrawal. The complexity, high risk, and busyness of clinical work can lead to stress among nurses, influencing the occurrence of work withdrawal behaviors. In our study, the majority of nurses reported experiencing moderate to high levels of work stress, and higher stress levels were associated with increased work withdrawal behaviors, as validated in prior studies (Liu et al., 2022). Additionally, factors such as nurses’ literacy, years of experience, and knowledge and skill acquisition also play a significant role in the occurrence of work withdrawal behaviors. Nurses with higher levels of education and extensive clinical experience tend to exhibit greater work engagement, thereby enhancing the efficiency and quality of care; conversely, lower education and experience negatively impact nurses’ motivation and the quality of care (Almarwani and Alzahrani, 2023). Therefore, interventions such as intra-hospital training and skill competitions should be implemented to improve nurses’ competency and reduce work withdrawal behaviors.

Our study found that feelings of underqualification were positively associated with work withdrawal behavior, accounting for a direct effect of 45.3% of the total effect. As the field of nursing continues to develop, the research capabilities required of nurses in clinical settings have become increasingly demanding. However, many clinical nurses today lack access to necessary resources for professional development and often show reluctance to pursue further education. These challenges, combined with the demands of research work, negatively impact job satisfaction and contribute to work withdrawal behaviors (Hakvoort et al., 2022). In contrast, nurses who actively participate in training and research studies exhibit greater innovation and are better equipped to meet job demands, which in turn enhances their commitment to work and reduces work withdrawal behaviors (Song et al., 2023). Therefore, beyond improving their own skills, hospitals should also proactively provide resources and opportunities for professional growth, creating a win-win situation for both the organization and its employees.

As we hypothesized, self-disgust mediated the relationship between feelings of underqualification and work withdrawal behavior, accounting for 24.7% of the total effect. In other words, nurses’ feelings of underqualification may lead to self-disgust, which influences their work withdrawal behaviors. Research indicates that unpleasant sensory stimuli encountered by nursing students in clinical settings, along with poor management of nurse–patient relationships and unclear job responsibilities, can result in higher levels of self-disgust, diminished confidence in nursing practice, and increased turnover rates in the profession (Cho et al., 2023). Similarly, clinical nurses with high aversion sensitivity often lack the skills to address problems effectively, tend to adopt avoidant behaviors, and show a positive correlation with their willingness to leave the profession (Song et al., 2024). Therefore, it is crucial to enhance nurses’ problem-solving skills and promote humanistic care, while hospitals should implement personalized interventions aimed at reducing self-disgust and improving nurses’ overall well-being at work.

Consistent with our hypothesis, self-efficacy mediated the relationship between feelings of underqualification and work withdrawal behavior, accounting for 21.2% of the total effect. In other words, feelings of underqualification can reduce work withdrawal behaviors by increasing self-efficacy. Prior research has shown that self-efficacy can indirectly diminish work withdrawal behaviors in nurses by moderating the relationship between strengths utilization and work engagement (Chu et al., 2022). Nurses with low self-efficacy are more vulnerable to the negative effects of stressors, including anxiety and depression, which can affect mental health, diminish well-being at work, and lead to work withdrawal (Lu et al., 2023). One study found that a blended simulation mastery learning intervention quickly improved novice nurses’ clinical skills, enhanced their self-efficacy in patient care, and boosted job confidence (Takashiki et al., 2023).

Furthermore, this study found that self-disgust and self-efficacy mediated the relationship between feelings of under-qualification and work withdrawal behavior, accounting for 9.1% of the total effect. The increased feelings of underqualification among nurses may contribute to heightened feelings of self-disgust; however, reducing self-disgust and enhancing self-efficacy can mitigate burnout and decrease the likelihood of work withdrawal behaviors. One study demonstrated that nurses in Pakistan improved the quality of care by overcoming negative emotions and bolstering self-efficacy, which enhanced their cross-cultural communication skills and ability to manage their emotions effectively (Furman et al., 2021). Additionally, when new nurses face multiple stressors, including lack of job competence, exam pressure, and poor interpersonal communication, they can engage in positive self-regulation and adaptation strategies, which help reduce negative emotions such as anxiety, aversion, and fear, thereby decreasing work fatigue and increasing work engagement (Han et al., 2022). These findings align with our results, suggesting that nurses’ self-efficacy can be enhanced through positive interventions, ultimately mitigating various negative impacts associated with clinical work.

## 5 Limitations

This study has several limitations. First, the cross-sectional design restricts the ability to draw causal inferences between the variables of interest; therefore, future longitudinal studies could be conducted to address this gap in causality. Second, the use of convenience sampling from a single province in China may introduce sampling bias, limiting the generalizability of the findings; multicenter studies in the future could help validate these results. Third, this study did not categorize the sample of nurses further; future research could establish subgroups based on seniority, education level, hospital level, and department to analyze the incidence of work withdrawal behaviors and the factors influencing them. Fourth, the self-efficacy scale is universal and lacks specificity for nurses. Specific scales can be developed and applied for research in the future. This study focused solely on the positive aspect of self-efficacy, without exploring the effects of external factors, such as social support, on the relevant variables. Future research should consider investigating the mediating role of both self-efficacy and external support in the relationship between feelings of under-qualification and work withdrawal behaviors. Furthermore, the work withdrawal behavior scale includes relatively straightforward items that may trigger avoidance psychology among nurses. When combined with social expectation bias, this can lead nurses to provide idealized responses, resulting in potential deviations in the findings. To mitigate research bias in the future, it would be beneficial to employ more objective research methods, such as psychological experiments, or to quantify work withdrawal behaviors. Finally, this study did not propose specific interventions to mitigate work withdrawal behaviors; this could be a valuable area for further exploration.

## 6 Conclusion

The results of this study indicated that nurses’ work withdrawal behaviors were at a moderately low level. According to the stress response model, feelings of under-qualification can have a direct impact on work withdrawal behavior, demonstrating a significant positive correlation. Self-disgust and self-efficacy have mediating effects on the lack of qualifications and work withdrawal behavior. As a positive factor, self-efficacy can effectively reduce the sense of inadequacy and self-disgust, and reduce the occurrence of work withdrawal behavior. From the perspective of nursing research, nurses can enhance their scientific research theories and professional skills by attending academic conferences and specialized training. They can learn psychiatric nursing, master relevant psychological support methods, reduce feelings of self-disgust at work, and better serve patients from a humanistic nursing perspective. The hospital has established a specialized psychological support system that regularly conducts psychological counseling and mental health lectures. Nursing management provides nurses with the latest clinical nursing knowledge and mental health theories in a timely manner and ensures appropriate scheduling to meet their needs. From a nursing practice perspective, Hospitals should provide ample opportunities for further study and attendance at academic conferences to help nurses enhance their professional skills. In addition, enhancing the physical environment by providing a relaxing break room for decompression and implementing appropriate psychological activities—such as mindfulness-based stress reduction, yoga, and Baduanjin—can help alleviate nurses’ negative emotions. Nursing managers can listen to nurses’ needs and attentively monitor their emotional states by conducting informal discussions or allowing anonymous emotional emails. Nurses should maintain a positive learning attitude, enhance their clinical skills, and manage their emotions through active meditation, keeping an emotional diary, and other methods. They should also take the initiative to seek psychological help and maintain a healthy lifestyle. To improve the mental health of nurses from the above two aspects, so as to enhance the self-efficacy, ensure the quality of clinical nursing, and reduce the occurrence of work withdrawal behavior.

## Data Availability

The original contributions presented in the study are included in the article/supplementary material, further inquiries can be directed to the corresponding author/s.
